# Comment on “Role of meditation on the essence of self in the psychological profile, quality of life and lifestyle – a comparative study”

**DOI:** 10.1016/j.clinsp.2025.100719

**Published:** 2025-07-14

**Authors:** Zeeshan Solangi, Rachana Mehta, Ranjana Sah

**Affiliations:** a Department of Medicine, BMC South, BMC Health System, MA, USA; b Yale University, School of Medicine, New Haven, CT, USA; c Clinical Microbiology, RDC, Manav Rachna International Institute of Research and Studies, Faridabad, Haryana, 121004, India; d Department of Paediatrics, Dr. D. Y. Patil Medical College Hospital and Research Centre, Dr. D. Y. Patil Vidyapeeth (Deemed-to-be-University), Pimpri, Pune, 411018, Maharashtra, India; e Department of Public Health Dentistry, Dr. D. Y. Patil Dental College Hospital and Research Centre, Dr. D. Y. Patil Vidyapeeth (Deemed-to-be-University), Pimpri, Pune, 411018, Maharashtra, India

Dear Editor,

We read with great interest the study by Arantes et al., which explores the psychosocial correlates of Gurusakāsha, a third-phase meditative practice rooted in tantric philosophy, using a battery of validated psychological and lifestyle scales.^1^ While the authors provide an important starting point in the investigation of lesser-known meditative modalities beyond mainstream mindfulness, several conceptual and methodological limitations warrant careful scrutiny before extrapolating these findings toward broader applications.

First, the decision to employ a cross-sectional, observational design inherently restricts the ability to infer causality between Gurusakāsha meditation and the observed psychological outcomes. Although the authors acknowledge this limitation, they do not sufficiently account for the risk of reverse causation: individuals with inherently higher resilience and lower stress may be more inclined to sustain long-term meditative practice, leading to selection bias.^2^ Furthermore, the sample included a relatively homogeneous group of long-time practitioners from a single institutional lineage (Instituto Visão Futuro), raising concerns about cultural clustering, spiritual indoctrination bias, and potential confounding by non-meditation lifestyle factors such as yoga or vegetarianism.

Second, the imbalance in group size and the modest total sample (*n* = 52) significantly undermines the statistical power of the study, particularly in subgroup analyses. While matching for age, gender, and education is methodologically sound, the Meditation Experts (ME) group had substantially higher rates of physical activity, which is itself a well-established mediator of improved resilience, mood, and quality of life.^3^

The authors report this difference yet fail to adjust for it in multivariate models, compromising the attribution of outcomes solely to meditation practice.

Third, the application of certain psychometric tools raises reliability concerns that the authors only partially address. The Visual Analogue Mood Scale (VAMS) scale exhibited a negative Cronbach’s alpha, rendering its findings non-interpretable. Instead of discarding or replacing the tool with a more psychometrically stable instrument, the authors attempt post-hoc rationalization linked to participant travel stress, which remains speculative. Similarly, the Interpersonal Reactivity Index of Davis (IRI) showed only borderline reliability (α = 0.65), and differences in emotional subscale interpretations between tantric philosophy and Western constructs were posited as explanatory. This introduces a philosophical relativism that undermines standardized cross-group psychological comparisons. If standard empathy scales are deemed culturally incongruent, this limitation should have been anticipated and corrected through either adaptation or alternative inventories rooted in Eastern contemplative traditions.

Additionally, while the Individual Lifestyle Profile (PEVI) scale data suggest lifestyle advantages among meditators, the lack of longitudinal tracking or behavioral diaries to document daily routines, dietary intake, or practice fidelity limits the internal validity of these claims. The influence of dietary restriction (e.g., lacto-vegetarianism) or communal reinforcement through guru-centric narratives may independently contribute to lifestyle and psychosocial outcomes, distinct from meditation per se.^4^^,^^5^

Lastly, the study's theoretical framework remains anchored in tantric symbolism (e.g., visualization of a lotus at the cranial vertex), yet it does not attempt to operationalize or quantify the meditative components (mantra repetition, visual imagery, breath regulation) in a reproducible or neuroscientific manner. Given the reference to concurrent fMRI data (unpublished), it is a missed opportunity not to correlate objective neural measures with psychological indices, especially since brain-based models of meditation are now central to contemplative neuroscience.^6^, ^7^, ^8^

In conclusion, while this study courageously investigates a non-mainstream contemplative practice, it falls short of substantiating its claims due to limitations in design, measurement, and analytic control. Future research must incorporate prospective longitudinal designs, active control arms, mechanistic biomarkers, and culturally sensitive tools to truly discern the distinct contributions of Gurusakāsha to psychological and lifestyle outcomes.

## Ethical approval

Not required.

## Clinical trial registration details/Number

Not applicable, as this study does not report a clinical trial.

## Research registry number

Not applicable.

## Human ethics and consent to participate declarations

Not applicable, as no patient data were collected or analyzed in this study.

## Generative AI use statement

Generative AI tools, including Paperpal and ChatGPT-4o, were utilized solely for language, grammar, and stylistic refinement. These tools had no role in the conceptualization, data analysis, interpretation of results, or substantive content development of this manuscript. All intellectual contributions, data analysis, and scientific interpretations remain the sole work of the authors. The final content was critically reviewed and edited to ensure accuracy and originality. The authors take full responsibility for the accuracy, originality, and integrity of the work presented.

## CRediT authorship contribution statement

**Zeeshan Solangi:** Conceptualization, Methodology, Validation, Supervision, Project administration, Writing – original draft, Writing – review & editing. **Rachana Mehta:** Writing – original draft, Writing – review & editing. **Ranjana Sah:** Writing – original draft, Writing – review & editing.

## Declaration of competing interest

The authors declare no financial interests relevant to this study.

## Data Availability

Not applicable, as no data were generated or analyzed in this study.
